# Highly selective colorimetric detection and preconcentration of Bi(III) ions by dithizone complexes anchored onto mesoporous TiO_2_

**DOI:** 10.1186/1556-276X-9-62

**Published:** 2014-02-06

**Authors:** Mohd Faisal, Adel A Ismail, Farid A Harraz, Houcine Bouzid, Saleh A Al-Sayari, Ali Al-Hajry

**Affiliations:** 1 Promising Centre for Sensors and Electronic Devices (PCSED), Advanced Materials and Nano-Research Centre, Najran University, P.O. Box 1988, Najran 11001, Saudi Arabia; 2 Nanostructured Materials and Nanotechnology Division, Central Metallurgical Research and Development Institute (CMRDI), P.O. Box 87, Helwan, Cairo 11421, Egypt; 3 College of Science and Arts-Sharoura, Najran University, Sharoura, Saudi Arabia; 4 Department of Physics, College of Science and Arts, Najran University, P.O. Box 1988, Najran 11001, Saudi Arabia

**Keywords:** Mesoporous TiO_2_, Optical sensor, Detection, Preconcentration, Bi(III) ions

## Abstract

We successfully developed a single-step detection and removal unit for Bi(III) ions based on dithizone (DZ) anchored on mesoporous TiO_2_ with rapid colorometric response and high selectivity for the first time. [(DZ)_3_-Bi] complex is easily separated and collected by mesoporous TiO_2_ as adsorbent and preconcentrator without any color change of the produced complex onto the surface of mesoporous TiO_2_ (TiO_2_-[(DZ)_3_-Bi]) at different Bi(III) concentrations. This is because highly potent mesoporous TiO_2_ architecture provides proficient channeling or movement of Bi(III) ions for efficient binding of metal ion, and the simultaneous excellent adsorbing nature of mesoporous TiO_2_ provides an extra plane for the removal of metal ions.

## Background

Bi(III) ion in the environment is highly fatal to human beings and in particular to aquatic species in seawater. The development of solely selective, separation, preconcentration, and detection method for Bi(III) ions at ultratraces is a challenging task because of their very low concentrations in natural samples and strong interference from the real sample matrices. Thus, in recent years, considerable attention has been focused on the preconcentration and/or monitoring of ultratrace Bi(III) ions [1]. Solid phase extraction techniques have provided excellent alternative approach to liquid-liquid extraction for Bi(III) preconcentration prior to analyte determination step [2-4]. Several supporters such as silica [5-7], clays [8], biomass [9], resins [10,11], and carbons [12,13] have been modified with chelating groups for the adsorption of heavy metal ions. In our previous work, first molecular receptors were anchored onto mesoporous silica and then this framework was used for the detection of metal ions [14-21]. However, few reports are available for the detection of heavy metals using TiO_2_ films [22,23]. Nanocrystalline TiO_2_ films were employed for naked-eye colorimetric detection of mercury in aqueous solution using N719 dye (N719 = *bis*(2,2A-bipyridyl-4,4A-dicarboxylato) ruthenium(II) *bis*(tetrabutylammonium) *bis*(thiocyanate)) [22,23]. Mesoporous TiO_2_ is supposed to be a potentially active material for designing optical sensor due to its excellent surface area and high optical transparency in the visible part of the spectrum [22]. When mesoporous TiO_2_ is dispersed in water, then the surface becomes anionic in nature and increases in surface area that would render the more coverage of hydroxyl groups (OH) from H_2_O [24]. In sensing application, mesoporosity provides the desired high accessible surface and easier movement of metal ions for efficient binding; simultaneously excellent adsorbing properties of mesoporous TiO_2_ provide an extra plane for removal of metal ion. In this contribution, we successfully detect and preconcentrate Bi(III) ion in a single step using mesoporous TiO_2_ without any color change of the produced complex [(DZ)_3_-Bi] onto the surface of mesoporous TiO_2_ {TiO_2_-[(DZ)_3_-Bi]} at different Bi(III) concentrations. To the best of our knowledge, this is the first report briefing the single-step detection and removal of Bi(III) ions utilizing mesoporous TiO_2_.

## Methods

### Materials

The block copolymer surfactant EO_106_-PO_70_EO_106_(F-127,EO = -CH_2_CH_2_O–,PO = -CH_2_(CH_3_)CHO–), MW (12,600 g/mol), Ti(OC(CH_3_)_3_)_4_ (TBOT), HCl, CH_3_OH, C_2_H_5_OH, CH_3_COOH, and dithizone were purchased from Sigma-Aldrich (St. Louis, MO, USA).

### Preparation of mesoporous TiO_2_

Mesoporous TiO_2_ nanocrystals were synthesized through a simple one-step sol–gel process in the presence of the F127 triblock copolymer as a structure-directing agent. To minimize possible variables, the molar ratio of each reagent in the starting solution was fixed at TiO_2_/F127/C_2_H_5_OH/HCl/CH_3_COOH = 1:0.02:50:2.25:3.75. In particular, 1.6 g of F127, 2.3 mL of CH_3_COOH, and 0.74 mL of HCl were dissolved in 30 ml of ethanol and then added to 3.5 ml of TBOT [25]. The mixture was stirred vigorously for 60 min and transferred into a Petri dish. Ethanol was subsequently evaporated at 40°C, and a relative humidity of 40% for 12 h was set followed by the transfer of the sample into a 65°C oven and ageing for an additional 24 h. The as-made mesostructured hybrids were calcined at 450°C in air for 4 h at a heating rate of 1°C/min and a cooling rate of 2°C/min to remove the surfactant and to obtain the mesostructured TiO_2_.

### Characterization

Transmission electron microscopy (TEM) was conducted at 200 kV with a JEOL JEM-2100 F-UHR field-emission instrument (Tokyo, Japan) equipped with a Gatan GIF 2001 energy filter (Pleasanton, CA, USA) and a 1 K CCD camera in order to obtain EEL spectra. Field emission scanning electron microscope (FE-SEM) images were carried out with a FE scanning electron microanalyzer (JEOL-6300 F, 5 kV). X-ray diffraction (XRD) data were acquired on a PANalytical X’ port diffractometer using CuKα_1/2_, *λ*α_1_ = 154.060-pm and *λ*α_2_ = 154.439-pm radiation. Raman spectroscopy was carried out using a Perkin Elmer Raman Station 400 (Waltham, MA, USA). The nitrogen adsorption and desorption isotherms were measured at 77 K using a Quantachrome Autosorb 3B after the samples were vacuum-dried at 200°C overnight. The sorption data were analyzed using the Barrett-Joyner-Halenda (BJH) model with Halsey equation [26]. Fourier transform infrared spectroscopy (FTIR) spectra were recorded with a Bruker FRA 106 spectrometer (Ettlingen, Germany) using the standard KBr pellet method. Reflectance spectrum was taken at room temperature using UV-visible spectrophotometer (lambda 950 Perkin Elmer) fitted with universal reflectance accessory in the range of 200 to 800 nm and using BaSO_4_ as reference.

### Bi(III) ion detection

The solutions of different concentrations of Bi(III) ions ranging from 0.001 to 1 ppm were prepared in a buffer solution of pH 4. The working solution of DZ was prepared by dissolving 10 mg of dithizone in 100 ml of ethanol. The buffer solution of 0.2 M KCl-HCl of pH 2, 0.1 M CH_3_COOH–CH_3_COONa of pH 4, sodium dihydrogen phosphate and disodium hydrogen phosphate solution of pH 7, and 0.1 M disodium hydrogen phosphate-HCl of pH 9 was used to study the effect of pH on the adsorption of the Bi(III) ions on the designed nanosensors. A series of experiments has been carried out for the different concentrations of Bi(III) ions ranging from 0.001 to 100 ppm. For the detection of the metal ions, 5 mg of mesoporous TiO_2_ was constantly stirred in 20 ml of metal-ion solution of desired pH for 5 min to achieve the heterogeneous solution. One milliliter ethanolic solution of DZ was added to the above solution at room temperature with constant stirring for 1 min. The solution was then filtered using Whatmann filter. The filtrate was then analyzed for metal ion and absorbance using UV-visible spectrophotometer (lambda 950 Perkin Elmer). Bi(III) sorption took place quantitatively as indicated from the analysis of the Bi(III) ions in effluent solutions by ICP-OES. After extraction, the ultratrace concentrations of the remained ions in the test aqueous solutions were estimated by ICP-MS. Also, the TiO_2_-DZ-Bi complex was analyzed by UV-visible diffuse reflectance spectra by collecting the material from Whatmann filter. Reflectance spectrum was taken at room temperature using UV-visible spectrophotometer (lambda 950 Perkin Elmer) fitted with universal reflectance accessory in the range of 200 to 800 nm.

## Results and discussion

The prepared mesoporous TiO_2_, TiO_2_-DZ, and TiO_2_-[(DZ)_3_-Bi] have been investigated. XRD pattern reflections from anatase phases with peaks characteristic for the (101), (004), (200), (211), and (213) lattice planes evince that TiO_2_ phase easily nucleates during heating and subsequently transforms into nanocrystals upon calcination at 450°C (see Additional file 1: Figure S1). Even upon the addition of DZ anchored on the mesoporous TiO_2_ (Additional file 1: Figure S1, curve b) and after the (Bi(DZ)_3_) complex was collected onto the surface of mesoporous TiO_2_, the intensity of the mean peak (101) for all the samples was similar and there is no significant change in the crystallinity of the TiO_2_ anatase phases. Nitrogen adsorption isotherms of the TiO_2_ mesoporous and TiO_2_-DZ are investigated (see Additional file 2: Figure S2). Typical reversible type-IV adsorption isotherms are found for both samples. The sharpness of the inflection resulting from capillary condensation at relative pressures *p/p*_0_ between 0.45 and 0.7 is characteristic for mesostructures. The mesoporous TiO_2_ possesses high surface areas of 174 m^2^ g^-1^ and large pore volumes of 0.29 cm^3^ g^-1^ at 450°C; they are reduced to 143 m^2^ g^-1^ and 0.22 cm^3^ g^-1^, respectively, as a result of the DZ probe anchoring the pores. Also, the pore diameter is slightly decreased from 8.11 to 6.3 nm; this further confirms the DZ probe anchoring the pores.

**Figure 1:**
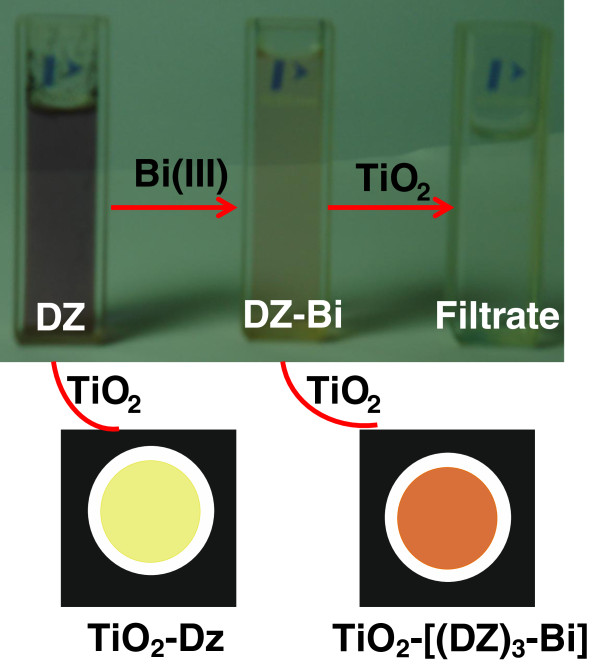
**Sensing mechanism based on binding 0.5-ppm solution of Bi(III) ion with organic chromospheres (DZ) in solution-phase.** The binding led to color change which corresponds to the formation of complex between the Bi(III) ion and DZ, and the final interaction of the formed complex with the mesoporous TiO_2_ led to the formation of highly stable TiO_2_-[(DZ)_3_-Bi] complex.

For the first time, we have successfully designed a highly sensitive novel sensing system and preconcentrator based on mesoporous TiO_2_. Small particles and large surface area of mesoporous TiO_2_ play an important role in terms of accessibility and adsorption amount. These characteristic features of sensing system increase the possibility of binding events or complex formation between metal ions and sensor, as clearly shown by our results in which the TiO_2_/DZ-based nanosensor shows excellent sensing performance at ultratrace level of concentrations and also the simultaneous removal of Bi(III) ions (Figure 1). The mechanism based on binding of the Bi(III) ion with organic chromospheres (DZ) in the solution phase led to color change which corresponds to the formation of complex between Bi(III) ion and DZ, and the final interaction of the formed complex with mesoporous TiO_2_ led to the formation of stable TiO_2_-[(DZ)_3_-Bi] complex which can be easily separated by simple filtration, leaving behind clear transparent filtrate (Figure 1). The sensing system responds very fast regardless of Bi(III) concentration and demonstrates color change only in few seconds. Furthermore, the designed sensor completely removed the color complex without any leaching, leaving a colorless and transparent filtrate, suggesting the stable binding between the mesoporous TiO_2_ and [(DZ)_3_-Bi] complex and also the complete removal of Bi(III) ions (Figure 1).

**Figure 2:**
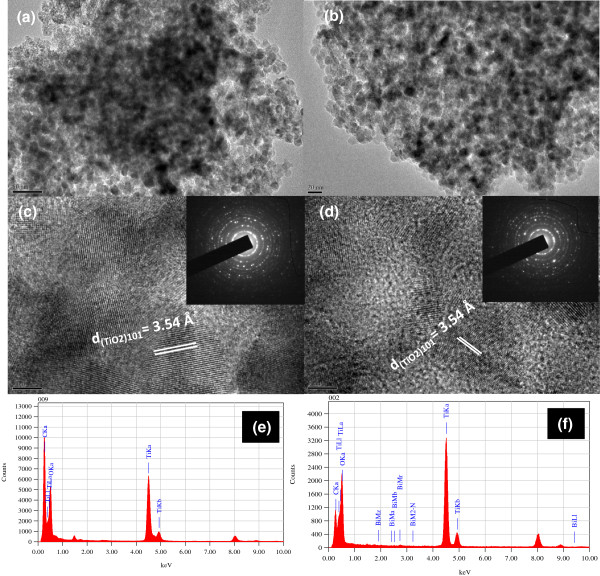
**TEM and HRTEM images and EDS analysis of the samples.** TEM images of TiO_2_-DZ **(a)** and TiO_2_-[(DZ)_3_-Bi] **(b)** samples. HRTEM images of TiO_2_-DZ **(c)** and TiO_2_-[(DZ)_3_-Bi] **(d)**. The EDS analysis of TiO_2_-DZ **(e)** and TiO_2_-[(DZ)_3_-Bi] complex **(f)**.

The TEM images of the TiO_2_-DZ and TiO_2_-[(DZ)_3_-Bi] samples were investigated (Figure 2). It is clearly seen that all the particles are spherical in shape with a uniform size distribution. Interestingly, there is no change in the shape and uniformity of TiO_2_ after anchoring the DZ probe (TiO_2_-DZ) and even TiO_2_-[(DZ)_3_-Bi] complex (Figure 2a,b). The TEM images indicated that the prepared TiO_2_ was mesoporous in nature (Figure 2a,b). The particle size of the TiO_2_ nanocrystals has been measured to be appropriately 10 nm. As seen in the HRTEM images (Figure 2c,d), the atomic planes of the TiO_2_ particles are separated by 3.54 Å, which agrees with the (101). It is important to note that the incorporation of either DZ or [(DZ)_3_-Bi] complex into the TiO_2_ framework does not have an effect on the mesostructure. The selected area electron diffraction (SAED) pattern (Figure 2c,d inset) further confirms that the TiO_2_ anatase is formed. The EDS analysis showed that there is a representative EDS pattern of the TiO_2_-DZ (Figure 2e) and even TiO_2_-[(DZ)_3_-Bi] complex (Figure 2f), and it revealed the presence of Ti, O, and C elements in the obtained TiO_2_-DZ; however, the Ti, O, C, and Bi elements were detected in the TiO_2_-[(DZ)_3_-Bi] sample. This indicated that the DZ probe is anchored onto the TiO_2_ network, and in the case of TiO_2_-[(DZ)_3_-Bi], Bi is observed as well; this further confirms that the [(DZ)_3_-Bi] complex was formed into the TiO_2_ pores. The FTIR spectra for the meso-TiO_2_, TiO_2_-DZ, and TiO_2_-DZ-Bi samples revealed a broad absorbance peak in the range from 3,100 to 3,450 cm^-1^ assigned to hydroxyl vibration and a strong absorbance peak around 1,628 cm^-1^ attributed to the vibrations of the surface-adsorbed H_2_O and Ti-OH bonds (see Additional file 3: Figure S3). Also, after anchoring DZ, as you see in either TiO_2_-DZ or TiO_2_-[(DZ)_3_-Bi] samples, the FTIR spectra show distinct absorption peaks at 1,435 cm^-1^ corresponding to the C = S stretching mode, while the peak shifts to 1,352 cm^-1^ for the TiO_2_-[(DZ)_3_-Bi] sample due to the introduction of Bi(III) in C = S-Bi [27]. In the TiO_2_-DZ and TiO_2_-[(DZ)_3_-Bi] samples, the absorption peaks at 1,540 cm^-1^ is attributed to the benzene ring stretching band, whereas in the spectrum of TiO_2_-[(DZ)_3_-Bi], the peaks shift to 1,523 cm^-1^ due to the formation of Bi-N bond in Bi-N-C_6_H_5_.

**Figure 3:**
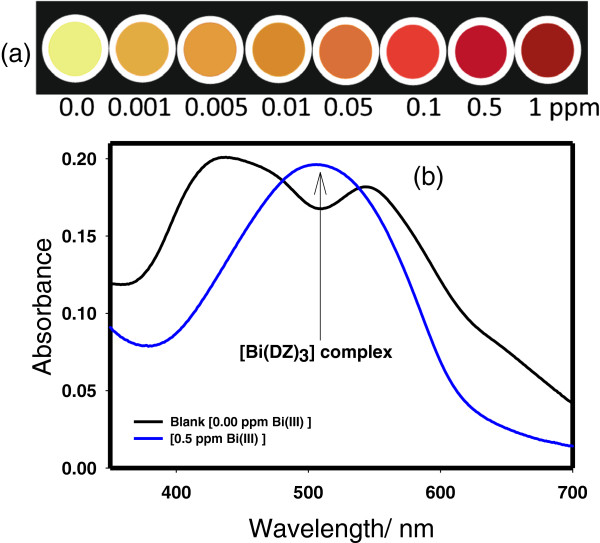
**Color changes and spectral patterns. (a)** The sequence of concentration-dependent changes in color of TiO_2_-DZ nanosensor after the detection of Bi(III) ions at different concentrations. **(b)** Spectral patterns obtained with DZ in the absence (blank) and in the presence of 0.5 ppm Bi(III) ions after 1-min reaction time at pH 4.

For the detection of Bi(III) ions, 5 mg of mesoporous TiO_2_ was constantly stirred in 20 ml of Bi(III) ion solution at different concentrations and pH value of 4 for 5 min to achieve the heterogeneous solution. One milliliter ethanolic solution of DZ was added to the above solution at room temperature, and the mixture was left to allow reaction for 1 min. Change in color can be easily distinguished by naked eye, and optical changes can be easily quantified by UV-visible spectroscopy. Wide range of Bi(III) ion concentrations (0.001 to 1 ppm) has been studied using UV spectroscopy. The designed nanosensor shows high sensing ability at trace-level concentration of Bi(III) ion, suggesting easier flow of Bi(III) ion over a wide range of concentrations (Figure 3a). Mesoporous TiO_2_-based sensing system can be utilized in two ways, as a chemosensor simply by visual inspection and simultaneously this potentially interesting material could also serve as preconcentrators to provide high adsorption efficiency to remove the toxic metal ions in a single step by a strong interaction between the TiO_2_ and the [(DZ)_3_-Bi] complex. Our designed sensor provides a simultaneous detection and removal of Bi(III) ions without the use of sophisticated instrument. The stability of the DZ probe with TiO_2_ can be assessed by the fact that there was no indication of elution of the probe molecules during the detection of Bi(III) ion. Transparent, clear filtrate obtained after filtration confirmed the firm integration of mesoporous TiO_2_ and Bi(DZ)_3_ complex and also the preconcentrator properties of the designed sensing system. Besides that, the addition of Bi(III) ion which led to a rapid color transformation provides a very simple, sensitive and selective detecting approach. As can be seen from Figure 3a, in the absence of Bi(III) ions, the color of the designed sensor is light yellow or mud but after the formation of the [Bi(DZ)_3_] complex, the color becomes light orange (at 0.001 ppm of Bi), indicating the presence of Bi in the formed complex at very low concentration of the Bi(III) ions. As the concentration of the Bi(III) ions increases, the intensity of the color also increases and becomes brick color at high concentration of the Bi(III) ions. The rapid color changing behavior of the newly developed sensing system upon the addition of the Bi(III) ions may be due the fact that highly potent mesoporous TiO_2_ architecture provides proficient channeling or movement of the Bi(III) ions for efficient binding of metal ion, and the simultaneous excellent adsorbing nature of the mesoporous TiO_2_ provides an extra plane for the removal of metal ions. Figure 3b shows the spectral patterns obtained with DZ-based sensor in the absence (blank) and in the presence of 0.5 ppm Bi(III) ions. As can be seen, in the absence of the Bi(III) ions, i.e., blank which shows an absorbance maxima at 434 and 580 nm. The shorter wavelength corresponds to thiol, and the longer wavelength corresponds to the thione group of DZ. On the other hand, with 0.5-ppm Bi(III) ion solution, a complex formation occurs, and a single band appears near to 502 nm which confirms the formation of the [Bi(DZ)_3_] complex [18-21]. The absorbance at 502 nm was used to calculate the concentration of the [Bi(DZ)_3_] complex. Table 1 shows the absorbance value at 502 nm for each concentration studied.

**Table 1:** Absorbance values at 502 nm for each concentration studied

**No.**	**Concentration of Bi(III) ions in ppm**	**Absorbance (a.u.)**
1	0.001	0.1735
2	0.005	0.1771
3	0.01	0.1842
4	0.05	0.188
5	0.1	0.1936
6	0.5	0.197
7	1.0	0.217

One of the major advantages of the current proposed sensing system is the selective sensing performance in the presence of interfering cations and anions even at 5,000-times-more concentration of the interfering components in comparison to Bi(III) ions (see Additional file 4: Table S1). Thus, the current approach presents a highly selective nanosensor for the efficient recognition of Bi(III) ions. To study the interfering effect of possible cations and anions which may present with Bi(III) ions in the surrounding environment or wastewater, we added the interfering cations and anions to a 0.5-ppm solution of Bi(III) ions in the presence of the proposed nanosensor at pH 4. To ensure the selective performance of our TiO_2_-based sensor, we carried out the experiments up to high tolerance concentration of interfering cations and anions. The results show no significant changes at very high concentrations in color pattern obtained after the addition of various types of interfering cations and anions, confirming the highly selective nature of this mesoporous TiO_2_-based sensor. Only Fe^+3^, Cr^+3^, and Hg^+^ cations show interfering effect at high concentrations, i.e., 100 ppm or above out of the several cations taken into consideration. In case of anions only, I^-^ shows slight color change at 250 ppm which is almost 5,000 times more than the Bi(III) ion concentration.

## Conclusions

In summary, a very simple sensing approach for one-step detection and collection of Bi(III) ions without the use of any sophisticated technique or further modification of mesoporous TiO_2_-based nanosensor is demonstrated, and the sensing results could be easily detected by naked eye. The detection limit for the Bi(III) ions using mesoporous TiO_2_-based sensor is estimated to be approximately 1 ppb. The results presented herein have important implications in the development of colorimetric sensors based on mesoporous TiO_2_ nanocrystals for the simple, swift, and selective detection of toxic metal ions in solution.

## Competing interests

The authors declare that they have no competing interests.

## Authors' contributions

All authors participated in the design of the study. MF, AI, and FH carried out all the experiments. HB measured and analyzed the data of TEM and XRD. MF, AI, and FH participated in analysis of the results and drafted the manuscript. All authors, especially SAS and AAH, provided comments/suggestions to revise it. All authors read and approved the final manuscript.

## Supplementary Material

Additional file 1 XRD patterns of the samples. Click here for file

Additional file 2 **N**_**2 **_**sorption isotherms and pore size distributions (inset) of the of the samples.** Click here for file

Additional file 3 FTIR spectra for all the samples. Click here for file

Additional file 4 Contains a table that summarizes the color trend obtained for various interfering cations and anions. Click here for file
