# Supplementary material for: Highly selective colorimetric detection and preconcentration of Bi(III) ions by dithizone complexes anchored onto mesoporous TiO2
Source: Nanoscale Res Lett. 2014 Feb 6;9(1):62. doi: 10.1186/1556-276X-9-62 (PMC3922967; doi:10.1186/1556-276X-9-62)
Supplement: Additional file 1 — XRD patterns of the samples. [file 1556-276X-9-62-S1.doc]

**Additional file 1**

Figure. S1 XRD patterns of the synthesized mesoporous TiO_2_(a), TiO_2_- DZ(b) and TiO_2_-DZ-Bi(c) samples. Shifted for sake of clarity.
