# Supplementary material for: Highly selective colorimetric detection and preconcentration of Bi(III) ions by dithizone complexes anchored onto mesoporous TiO2
Source: Nanoscale Res Lett. 2014 Feb 6;9(1):62. doi: 10.1186/1556-276X-9-62 (PMC3922967; doi:10.1186/1556-276X-9-62)
Supplement: Additional file 2 — N 2 sorption isotherms and pore size distributions (inset) of the of the samples. [file 1556-276X-9-62-S2.doc]

Figure S2. N_2_ sorption isotherms and pore size distributions (inset) of the of the synthesized mesoporous TiO_2_ and TiO_2_- DZ.
