# Supplementary material for: Highly selective colorimetric detection and preconcentration of Bi(III) ions by dithizone complexes anchored onto mesoporous TiO2
Source: Nanoscale Res Lett. 2014 Feb 6;9(1):62. doi: 10.1186/1556-276X-9-62 (PMC3922967; doi:10.1186/1556-276X-9-62)
Supplement: Additional file 3 — FTIR spectra for all the samples. [file 1556-276X-9-62-S3.doc]

Figure S3 FTIR spectra for mesoporous TiO_2_, TiO_2_-DZ and TiO_2_-DZ-Bi samples
