# Supplementary material for: Highly selective colorimetric detection and preconcentration of Bi(III) ions by dithizone complexes anchored onto mesoporous TiO2
Source: Nanoscale Res Lett. 2014 Feb 6;9(1):62. doi: 10.1186/1556-276X-9-62 (PMC3922967; doi:10.1186/1556-276X-9-62)
Supplement: Additional file 4 — Contains a table that summarizes the color trend obtained for various interfering cations and anions. [file 1556-276X-9-62-S4.doc]

Table S1 Summarized the color trend obtained for various interfering cations and anions to 0.5 ppm of Bi(III) ion solution (20 ml) at pH 4 using 5 mg of nanocrystalline TiO_2_ mesoporous.

| **Foreign Cation/Anions** | **10 ppm** | **100 ppm** | **250 ppm** |
| --- | --- | --- | --- |
| Al^+3^ | No interference | No interference | No interference |
| Hg^+^ | No interference | No interference | Slight change in color |
| Ni^+2^ | No interference | No interference | No interference |
| Pb^+2^ | No interference | No interference | No interference |
| Fe^+3^ | No interference | No interference | Mud color appearance |
| Cu^+2^ | No interference | No interference | No interference |
| Cd^+2^ | No interference | No interference | No interference |
| Co^+2^ | No interference | No interference | No interference |
| Cr^+3^ | No interference | color become light | color become Green |
| Mg^+2^ | No interference | No interference | No interference |
| NO_3_^-^ | No interference | No interference | No interference |
| Cl^-^ | No interference | No interference | No interference |
| F^-^ | No interference | No interference | No interference |
| Br^-^ | No interference | No interference | No interference |
| I^-^ | No interference | No interference | Color become light |
| CO_3_^-2^ | No interference | No interference | No interference |
| C_6_H_5_COO^-^ | No interference | No interference | No interference |
| Mg^+2^ | No interference | No interference | No interference |
| SO_4_^-2^ | No interference | No interference | No interference |
| CO_3_^-2^ | No interference | No interference | No interference |
| SDS | No interference | No interference | No interference |
| CH_3_COO^-^ | No interference | No interference | No interference |
